# Benchmarking Deep Networks for Predicting Residue-Specific Quality of Individual Protein Models in CASP11

**DOI:** 10.1038/srep19301

**Published:** 2016-01-14

**Authors:** Tong Liu, Yiheng Wang, Jesse Eickholt, Zheng Wang

**Affiliations:** 1 School of Computing, University of Southern Mississippi, 118 College Drive #5106, Hattiesburg, Mississippi 39406-0001; 2 Department of Computer Science, Central Michigan University, Pearce Hall 416, Mount Pleasant, Michigan 48859-0001.

## Abstract

Quality assessment of a protein model is to predict the absolute or relative quality of a protein model using computational methods before the native structure is available. Single-model methods only need one model as input and can predict the absolute residue-specific quality of an individual model. Here, we have developed four novel single-model methods (Wang_deep_1, Wang_deep_2, Wang_deep_3, and Wang_SVM) based on stacked denoising autoencoders (SdAs) and support vector machines (SVMs). We evaluated these four methods along with six other methods participating in CASP11 at the global and local levels using Pearson’s correlation coefficients and ROC analysis. As for residue-specific quality assessment, our four methods achieved better performance than most of the six other CASP11 methods in distinguishing the reliably modeled residues from the unreliable measured by ROC analysis; and our SdA-based method Wang_deep_1 has achieved the highest accuracy, 0.77, compared to SVM-based methods and our ensemble of an SVM and SdAs. However, we found that Wang_deep_2 and Wang_deep_3, both based on an ensemble of multiple SdAs and an SVM, performed slightly better than Wang_deep_1 in terms of ROC analysis, indicating that integrating an SVM with deep networks works well in terms of certain measurements.

Protein structures play an important role in determining protein functions and addressing various problems in biomedical research. Experimental methods for determining protein structures such as X-ray crystallography, however, are relatively costly and not applicable in some situations. Therefore, it is essential to develop computational software to predict protein tertiary structures (i.e., protein models) based on a protein’s primary amino acid sequence. Various computational tertiary structure prediction methods have been developed and subsequently a large number of models can be generated from these automated software pipelines. Before a model is used, its quality is usually assessed to confirm whether it is globally reliable or which portions of it are reliable (i.e., structurally similar to the native structure)^1^. This process is known as quality assessment (QA) of protein models and can include evaluating the relative or absolute quality of one or more protein models or identifying segments with good quality. Quality assessment (QA) of protein models is a key topic in the field of protein structure prediction.

The *a priori* quality assessment of a single protein model was introduced into the Critical Assessment of Techniques for Protein Structure Prediction (CASP) experiment as an independent category in 2006^2^. Since then, multiple QA methods have been developed and improved. In general, there are three major types of QA methods: clustering-based methods^3^,^4^,^5^, single-model methods^5^,^6^,^7^, and quasi-single methods^8^,^9^. With clustering-based methods, a set of protein models associated with the same protein sequence are taken as input and the relative quality score of each model can be computed through its pairwise structural alignment with other models. The scores generated in this way are usually termed relative quality scores. With single-model methods, only one model is needed as input and the output is usually the absolute quality score of the model or quality of every residue of the model. The quasi-single method is a hybrid of the first two methods. For example, a quasi-single method may first use a single-model method to predict the global quality score for each model in a pool, choose the top 5 models as reference models, and then uses a clustering-based method to predict the final global scores by superimposing each model on each of the reference models^8^. Among these three approaches, clustering-based methods have usually performed better in the CASP experiments^1^,^10^. To be effective, however, the cluster-based approach needs a large pool of models as input (which is not a problem for competitions like CASP) and is based on an assumption that the highest-quality model is the model that shares the most structural similarity to the other models. This is problematic in practice since scientists may only have one or two models available and are usually interested in the absolute quality of the models. Moreover, in some cases, the quality range of input models varies widely, which may make the preceding assumption incorrect^8^,^10^. On the other hand, single-model methods do not have these limitations and can output absolute quality scores globally (i.e., one score for the entire model) and locally (i.e., one score for each residue). The local score usually indicates the deviation of the residue-specific prediction with the native structure of the model and can be used to distinguish which part of the protein model is reliable^11^.

Although single-model methods can predict residue-specific quality scores of a single model, which is useful for appropriate model usage and structural refinement, to date not many single-model tools have been developed. ProQ2^6^ is a single-model method that uses support vector machines (SVMs) and inputs such as structural information, solvent accessibility surfaces, evolutionary information and profile weighting. SMOQ^7^ is also a SVM-based single-model predictor. Both methods can predict the absolute quality scores on a residue-specific basis and convert the local scores to a global score. DL-Pro^12^ is another method for protein quality assessment and uses deep learning to classify a protein model as good or bad (i.e., a binary classification). However, in many cases it is not enough to only provide a global score to assess the model of interest and residue-specific details should be given to evaluate the model. Our work presented here is partially similar to SMOQ. However, we developed three novel methods based on deep learning algorithms and used several new measures to assess our methods.

In this study, we present four novel single-model methods (Wang_deep_1, Wang_deep_2, Wang_deep_3, and Wang_SVM) to predict the absolute residue-specific quality of individual protein models. The first three use multiple stacked denoising autoencoders (SdAs) with various configurations in terms of the number of hidden layers and learning rates to predict residue-specific deviations for a single model. An autoencoder is a mathematical model that learns a representation of an input vector so that it can be used to reconstruct the input^13^ (i.e., an autoencoder learns to map the input to itself, often through a smaller dimensional space). Denoising autoencoders are used to reconstruct the inputs from corrupted versions and several can be stacked in series to create a Stacked Denoising Autoencoder (SdA)^14^,^15^. Specifically, Wang_deep_2 and Wang_deep_3 were developed based on a machine learning ensemble, integrating multiple SdAs with an SVM. Wang_deep_1 integrated 10 SdAs, whereas Wang_SVM was developed solely based on an SVM.

## Results

### Assessment of global quality predictions

For benchmarking global quality estimates, the global correlation coefficients (stage_1 and stage_2, separately) between GDT_TSs and the corresponding predicted global scores were calculated and are shown in Table 1. There are two general approaches to predict the global score of a model: (1) derive the predicted global score from local estimates (e.g., Wang_deep_1; for details see methodology section); and (2) predict the global score directly (e.g., MULTICOM-CLUSTER trained an SVM model to predict the global score of a model). It is clear that MULTICOM-CLUSTER is the best performer in both stages. Wang_deep_1 is the best of our four tools in stage_1, while Wang_SVM outperforms our other methods in stage_2. For the weighted mean of Pearson’s correlation per target (wmPMCC), the first eight methods in Table 1 performed almost equally well in stage_1, whereas all groups have a relatively lower value in stage_2. Table 1 also shows that the performance of each QA predictor in stage_1 is better than that in stage_2, indicating that the predictors can predict global model quality more accurately when the models of interest have evenly distributed quality.

We split the 55 CASP11 targets into three categories (alpha, beta or alpha-beta) based on their secondary structures. There were 3 proteins which only consisted of alpha helices, 3 proteins which only consisted of beta-sheets and 49 proteins containing both alpha helices and beta-sheets. This categorization was done to identify whether the performance of single-model methods is related to secondary structure variations. The global quality predictions of the three categories were evaluated separately. In Table 2, it is noticeable that the correlation coefficients on alpha-beta proteins are usually larger than those on alpha and beta proteins, although the reason might be the smaller number of alpha and beta proteins. Almost all methods have a lower overall correlation score in stage_2 for beta proteins, indicating that single-model methods do not seem well suited at predicting global quality of beta protein models. This might be because of the long-range interaction of beta sheets that increases the prediction difficulty.

We also separated the CASP11 targets by the availability of a structural template and evaluated QA predictors on groups of models for which a template could or could not be detected. There are 37 template-based modeling targets (i.e., TBM, templates are available) and seven free modeling targets (i.e., FM, no template available). Table 3 shows that all of the methods have better performance in terms of global score correlation coefficients for TBM proteins than for FM proteins, except for wmPMCC in stage_2.

### Assessment of local quality predictions

**Figure 1:**
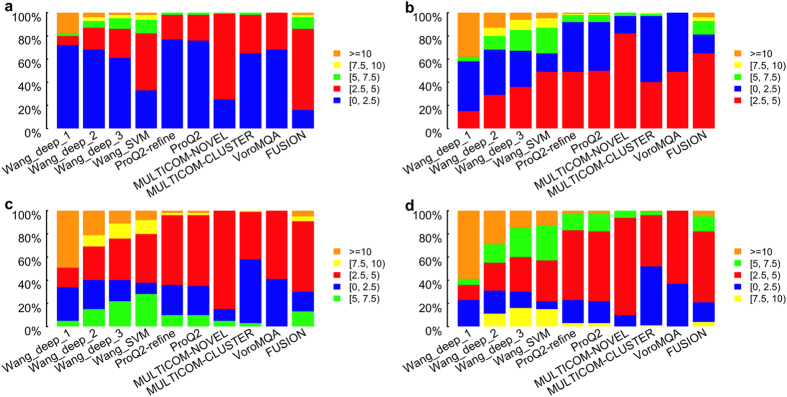
Local evaluation—the percentage of residues whose predicted deviations are within five different sets when the observed deviations of target residues belong to a range of (**a**) [0 Å, 2.5 Å), (b) [2.5 Å, 5 Å), (c) [5 Å, 7.5 Å), and (d) [7.5 Å, 10 Å), respectively.

Before benchmarking local estimates, we statistically analyzed the distributions of the predicted deviations of all structurally aligned residues whose observed deviations were in a certain range. For example, we at first selected the residues with *observed* deviations in the range 0–2.5 Å, and then computed the percentage of the residues whose *predicted* deviations are in the range of [0 Å, 2.5 Å), [2.5 Å, 5 Å), [5 Å, 7.5 Å), [7.5 Å, 10 Å) and [10 Å, ∞), respectively. Ideally, the percentage associated with the range of [0, 2.5 Å) should be 100% and all the other ranges should be zero. Figure 1 shows that our three methods, Wang_deep_2, Wang_deep_3, and Wang_SVM, have relatively better performance when the observed deviation is larger than 5 Å, whereas nearly all predicted deviations from the other predictors are within 5 Å no matter whether the observed deviations are greater than 5 Å.

**Figure 2:**
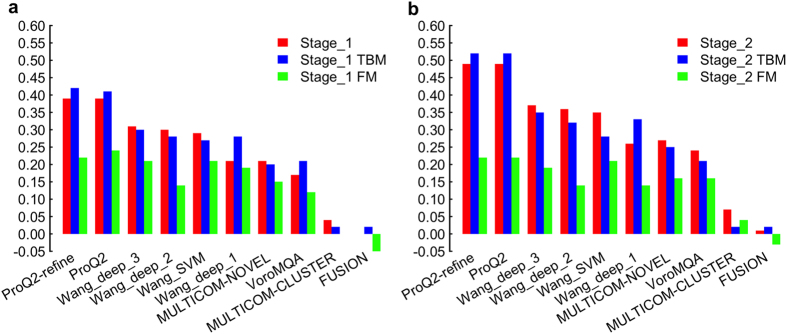
Local evaluation—the residue-specific prediction assessment by weight mean PMCC of all models in the pool of targets of interest, TBM proteins, and FM proteins separately.

To assess local estimates, we first computed the Pearson’s correlation between predicted and observed deviations of structurally aligned residues for all models for a target, and then transformed these Pearson’s correlation scores into a per-target value, the weighted mean of Pearson’s correlation (wmPMCC) as plotted in Fig. 2, showing that the ProQ group (ProQ2-refine and ProQ2) achieved the best performance followed by our four tools in both stages. It is obvious that the performance for every predictor in stage_2 is better than that in stage_1, which means single-model methods can have relatively better performance in predicting residue-specific deviations for models with higher quality. For TBM and FM proteins, almost every wmPMCC of the TBM proteins is higher than the corresponding values for FM proteins, which is consistent with the result in assessing global quality predictions.

**Figure 3:**
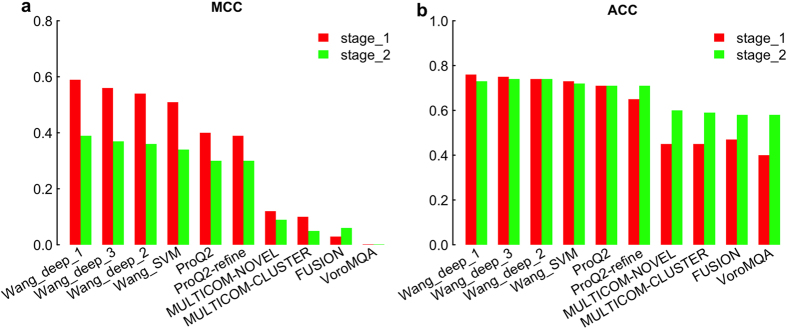
Local evaluation—the MCC and ACC when the threshold is set to 5 Å. An estimate is considered correct when both predicted and observed deviations are within 5 Å.

**Figure 4:**
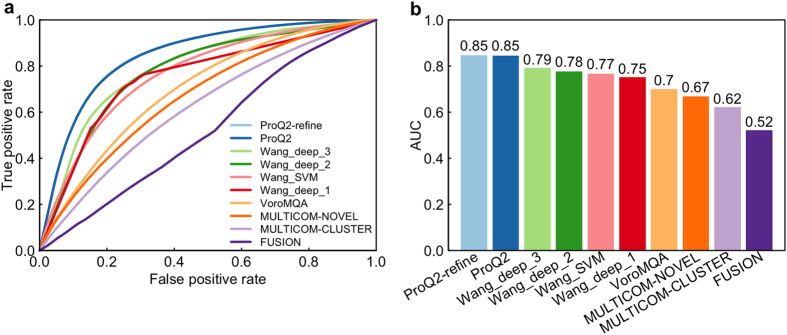
Local evaluation—the ROC analysis in stage_2 to assess the ability to identify reliable residues from unreliable residues. (**a**) The ROC curves for ten CASP11 QA tools and (**b**) the corresponding AUCs. Group names are sorted by their AUCs.

For assessing the ability to identify the reliable regions of an individual model, we used two measurements: MCC (Fig. 3) and ROC analysis (Fig. 4). Figure 3 shows that Wang_deep_1 achieved the highest scores in both stages followed by Wang_deep_3, Wang_deep_2, and Wang_SVM. Our four tools were also ranked above the other predictors in terms of ACC (Fig. 3(b)). As for ROC analysis, we only report the performance of all single-model predictors in stage_2, since the two stages yield similar results (data not shown); and with respect to this analysis, the ProQ group performs better than the other groups (Fig. 4(b)), followed by our methods.

### An example of local quality predictions

**Figure 5:**
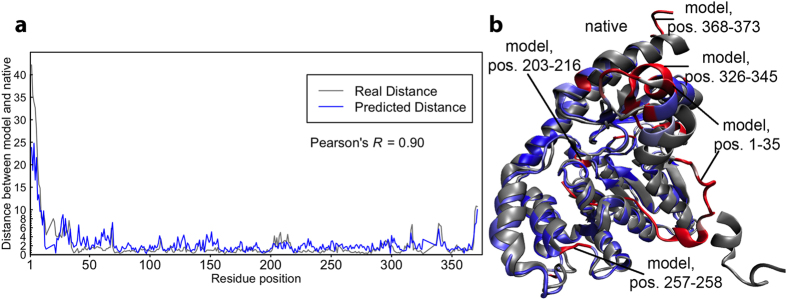
(**a**) an example illustrating the predicted deviations generated by Wang_deep_2 and the observed deviations from superimposing the experimental structure on the predicted model. The model in this example is “raghavagps-tsppred_TS3” for target T0819 in CASP11. (**b**) The visualization of the superimposition with the model in the color blue or red whereas red regions are the segments that have relatively larger real distances (>~3.5 Å) and blue for regions with relatively smaller real distances.

We have visualized a successful example illustrating the predicted residue-specific deviations generated by Wang_deep_2 and the corresponding observed deviations from superimposing the experimental structure on a CASP11 model (Fig. 5). The Pearson’s correlation between the predicted deviations and the observed deviations is 0.90; and the average absolute difference between the predicted and observed distance deviation is 1.32 Å. The superimposed model is shown in Fig. 5(b).

## Discussion

We have presented our four QA predictors using SVMs and deep learning algorithm SdAs and evaluated our four predictors and six other single-model methods participating in CASP11 at the global and local levels. This evaluation followed the official CASP assessment criteria by dividing the models for each target into two groups (i.e., selected 20 (stage_1) and best 150 (stage_2)) to identify whether the actual quality of a model is closely related to the performance of a method^10^.

At the global level, our methods perform relatively well in both stages, even though the global score we used is directly derived from residue-specific deviations whereas the best performer in this category applied an SVM model specifically trained for global quality predictions. We also found that the correlation coefficient in stage_1 for every group is larger than its respective value in stage_2, indicating that single-model methods can predict global quality more reliably if the global quality of the evaluated models is evenly distributed. The weighted mean of Pearson’s correlation per target provides further evidence for this discovery.

At the local level, the distributions of the predicted deviations demonstrated that the other methods almost always predict local scores in the range of 0–5 Å even when the real deviation is above 5 Å, whereas our three methods (Wang_deep_2, Wang_deep_3, and Wang_SVM) can predict many more deviations in the range of >5 Å when the real deviation is actually above 5 Å. Moreover, when the observed deviations are below 10 Å, Wang_deep_1 predicted many more residues with >10 Å than the other tools, indicating that it could be improved in future work. We found that all the QA predictors have a better weighted mean of Pearson’s correlation (wmPMCC) in stage_2 compared to stage_1. This may be caused by the fact that the observed deviations in stage_2 (i.e., best 150 models) are usually much lower than the ones in stage_1 (i.e., 20 selected models with evenly distributed quality) and most of the QA predictors have a bias towards making local predictions of less than 5 Å. As a result, models with better quality, that is, lower deviations, fit the bias of the QA predictors, causing a higher quality assessment in terms of accuracy. In terms of the ability to recognize reliable regions, our four methods performed better than the rest according to Matthew’s correlation coefficient, but slightly worse than the best predictor ProQ in terms of ROC analysis.

All in all, our methods, especially those based on SdAs, perform relatively well among the single-model methods participating in CASP11. The performance of Wang_deep_3 and Wang_deep_2 in distinguishing reliable residues indicates that combining SdAs and an SVM is a feasible and promising way to predict the absolute quality of a model. Furthermore, the better performance of Wang_deep_3 over Wang_deep_2 indicates that combining both original features with values generated from SdA output nodes is better than only inputting SdA outputted values into an SVM when combining SdAs with an SVM.

### Future work

In this version of the methods, we used the same amount of pre-training data for all the SdAs. It would be interesting to test different amounts of pre-training data and different parameters including pre-training epochs and number of hidden layers in order to benchmark the contribution of these parameters to the performance. We also plan to integrate additional software for predicting secondary structure and residue-residue contact as features as well as evaluate a larger sliding window size in order to capture non-local features.

## Methodology

### Test data set

To evaluate our methods, we selected 55 CASP11 targets excluding those without experimental structures or canceled by CASP11. Each of them has an experimental structure provided on the CASP website. Every QA predictor made predictions for two stages of models. In stage_1, 20 models evenly distributed in terms of model quality for each target were selected and presented to CASP11 participants for evaluation. In stage_2, the best 150 models per target were selected and presented for evaluation. In this way, the performance of QA predictors for diverse model quality could be found^10^.

In addition to our methods, we also evaluated six other single-model methods participating in CASP11, including FUSION, MULTICOM-NOVEL, MULTICOM-CLUSTER, ProQ2, ProQ2-refine, VoroMQA. For predicting local quality scores, there are four methods based on SVMs: MULTICOM-NOVEL, MULTICOM-CLUSTER, ProQ2, and ProQ2-refine. VoroMQA made use of knowledge-based potentials over inter-atomic contact areas and solvent contact areas to get the local deviation estimates^16^. FUSION used a probabilistic graphical model to calculate the likelihood of the local structure, and then converted it to a local quality score^16^. To predict global quality, eight methods (i.e., our four methods along with, FUSION, ProQ2, ProQ2-refine and VoroMQA) integrated their predicted local (residue-specific) scores into a global score^16^. MULTICOM-CLUSTER predicted global scores using an SVM model specifically trained to predict global scores and MULTICOM-NOVEL made a global quality prediction by combining global features of each model^16^. The QA predictions used in the evaluation of these 10 methods were downloaded from CASP website and constitute the estimates submitted by these methods during the CASP11 experiment.

### Measures of assessment

Sequence-dependent Local-Global Alignment (LGA)^17^ was used to superimpose a protein model with its experimental structure (i.e., native structure). After superimposition, LGA can generate a Global Distance Test Total Score (GDT_TS) and a set of C-α atoms’ Euclidean distances between two structurally aligned residues. GDT_TS is selected as the observed (real) score for benchmarking global quality estimates, as it is officially used by CASP.

The Pearson product-moment correlation coefficient (PMCC) was used to compute correlation coefficients between two vectors of scores, one from LGA (GDT_TSs or observed deviations) and the other from single-model methods (predicted global scores or deviations)^1^. Since it does not follow a normal distribution, the correlation coefficients should be transformed into an additive quantity before calculating their average value^2^. Fisher’s transformation (equation (1)) was used:


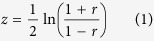


where r is Pearson’s correlation; z is the normally distributed variable transformed from r, having a standard error 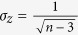 with n being the sample size. We use 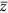 denoting the arithmetic mean score of a given set of z values. Finally, 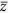 is inversely transformed into the weighted mean of Pearson’s correlation coefficient (wmPMCC) 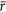 by using the inverse formula (equation (2))


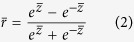


The receiver operating characteristic (ROC) analysis^18^ was chosen as a measure to assess the ability of QA groups to distinguish reliably predicted regions from unreliable regions. We also computed Matthews’s correlation coefficient (MCC)^19^ and Accuracy (ACC):


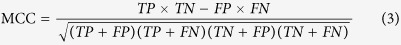



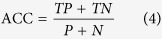


where *P* is the number of true positives (*TP*) plus false positives (*FP*), and *N* equals the number of true negatives (*TN*) plus false negatives (*FN*).

The predicted estimates from 10 CASP QA methods were evaluated at the global and local levels; and both levels were conducted for stage_1 and stage_2, separately. For benchmarking global predictions, we computed (1) the PMCC between predicted global scores and the corresponding real GDT_TSs of all models of all targets and (2) the wmPMCC in the units of each target’s PMCC using Fisher’s transformation as described above. Local assessments of residue-specific deviations were carried out in terms of the ability to identify reliable regions among all structurally aligned residues and whether the predicted deviations are closer to observed deviations. Specifically, the PMCC of residue-specific predicated deviations with observed deviations for each model was calculated and then all of the correlation coefficients were gathered to transform into a single value. Furthermore, the values of MCC and ACC for each group were computed. The threshold was set to 5 Å, which means if the observed distance and the corresponding predicted estimate are both below the threshold, it is considered a true positive. In the ROC analysis, we selected a set of thresholds: 1 Å up to 10 Å with an increment of 0.1 Å and each threshold is associated with a point on a ROC curve. The area under a ROC curve (AUC) indicates the classifier’s accuracy^20^.

### Overview and Features for machine learning

We developed four methods to predict residue-specific deviations between a predicted protein structure (model) and its native structure (native): Wang_SVM, Wang_deep_1, Wang_deep_2, and Wang_deep_3. All of the four methods used the same features as input:

1. Amino acid sequence: 20 bits with one bit as 1 and the others are 0 were used to encode 20 types of amino acids;
2. The difference of secondary structure as predicted from amino acid sequence by SSPRO^21^ compared to that parsed from the protein model by DSSP^22^. Specifically, if the predicted secondary structure is the same as the secondary structure parsed from model, it is labelled as a 1; otherwise, 0. In this way, every residue will be assigned either 0 or 1 for this feature;
3. The difference of solvent accessibility between that predicted from sequence by SSPRO and the values from the model as parsed by DSSP. This is similar to the secondary structure feature;
4. NNcon^23^ was used to predict the residue-residue contact probabilities based on amino acid sequence. This produced a probability for each pair of residues in the protein which indicated the likelihood of those residues having a Euclidean distance <= 8 Å in three-dimensional space. Based on the protein model, for each single residue, we selected all the other residues that have a sequential distance >= 6 residues away and with a Euclidean distance <= 8 Å in space. The probabilities of these pairs of residues as predicted by NNcon were averaged and used as a feature for the residue;
5. PSI-BLAST profile generated by PSI-BLAST. A PSI-BLAST profile provides evolutionary information collected from a family of similar protein sequences and can provide more information than the amino acid sequence. For each residue, the profiles for 20 amino acids were included as features;
6. The SOV (segment overlap measurement) score between the predicted secondary structure and secondary structure parsed from the model by DSSP. This is a global feature with one SOV score being generated for the whole model and was used for every residue in the model.

A 15-residue sliding window was applied to predict the deviation of a single residue, with seven residues ahead and seven after. The first models for every predictor (each CASP tertiary structure predictor is allowed to submit five models to CASP, we used the first model only) in both CASP8 and CASP9 were used to generate a benchmarking data set. Because we are dealing with residue-specific predictions, this generates about 1.2 million examples. All of our four methods were trained using this data set and blindly benchmarked in CASP11.

### Deep learning—Stacked Denoising Autoencoder

The deep learning architecture used in this study is a Stacked Denoising Autoencoder (SdA) based on Theano (http://deeplearning.net/software/theano/). An autoencoder is a mathematical model that learns a representation of an input vector so that it can be used to reconstruct the input^13^ (i.e., an autoencoder learns to map the input to itself, often through a smaller dimensional space). Denoising autoencoders are used to reconstruct the inputs from corrupted versions and several can be stacked in series to create a Stacked Denoising Autoencoder (SdA)^14^,^15^. The SdA training algorithm is composed of two steps of learning: unsupervised pre-training and supervised fine-tuning^14^. The first step is carried out by layers of denoising autoencoders.

For each layer, the original input *x-orig* is first corrupted to get the corrupted version of data 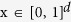; and we used a parameter called corruption level to control the level of corruption. Then, the corrupted input x is mapped to a hidden representation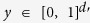 where 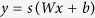 with *W* being the weighting matrix, *b* being the bias vector and the function 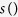 is the sigmoid function. Finally, y is mapped back to learn a reconstruction *z* by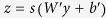, in which 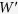
is the reconstruction weighting matrix, and 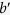 is the reconstruction bias. The Denoising Autoencoder is parameterized by the weight matrixes and bias vectors which are adjusted during training to minimize the cross-entropy of the reconstruction (equation (5)):


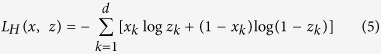


This step initiates the training process from the first layer, and then proceeds layer by layer. The (*m* **+ ***1)*^*th*^ layer would be trained by taking as input the representation of the data outputted from the *m*^th^ layer. When the unsupervised pre-tuning part is finished, all stacked layers of the denoising autoencoders are trained.

The supervised fine-tuning step can be taken after the unsupervised pre-tuning. A logistic regression model is added on top of the layers of denoising autoencoders (equation (6)):


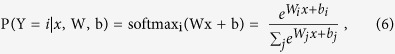


which calculates the probability of an input vector *x* belonging to the class *i* in the set of *Y*. *W* is the weighting matrix; *b* is the bias; and *j* can be any class in *Y*. After the probabilities of all available classes in *Y* have been calculated, an input vector *x* is predicted to the class having highest probability.

After adding the logistic regression layer, the entire learning structure (layers of denoising autoencoders plus logistic regression) is similar to an artificial neural network. However, its hidden layers share with the unsupervised pre-training the same number of layers and neurons in each layer; and the parameters of weight matrixes and bias vectors in hidden layers are the same as the ones trained in the unsupervised learning step. Label value *Y* is used as the target value to train the entire network (layers of denoising autoencoders plus logistic regression) by a backpropagation algorithm with logistic function as activation function. In this way, the training process makes the entire learning architecture fine-tuned, which means the parameters (all the weighting matrix *W* and bias *b*) in each hidden layer of denoising autoencoders and logistic regression model are further refined based on the class label *Y* of a training set.

### Design of machine learning architectures

We designed four learning architectures to be benchmarked in CASP11: Wang_SVM, Wang_deep_1, Wang_deep_2, and Wang_deep_3.

**Wang_SVM** uses a Support Vector Machine with RBF kernel trained using SVM-Light^24^.

**Figure 6:**
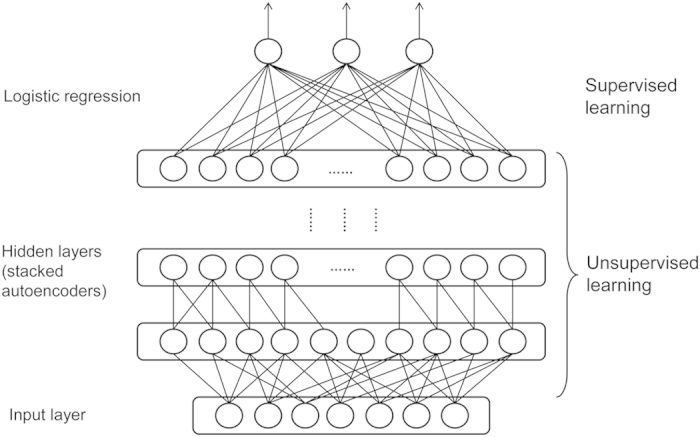
The architecture of stacked denoising autoencoders (SdAs).

**Wang_deep_1** integrated 10 SdAs, each of which has 20 output classes that represent different ranges of deviation. For example, class 1 represents that the deviation is between [0 Å, 0.25 Å), class 2 [0.25 Å, 0.5 Å), class 3 [0.5 Å, 0.75 Å), class 4 [0.75 Å, 1 Å), class 5 [1 Å, 1.25 Å), class 6 [1.25 Å, 1.5 Å), class 7 [1.5 Å, 1.75 Å), class 8 [1.75 Å, 2 Å), class 9 [2 Å, 2.5 Å), class 10 [2.5 Å, 3 Å), class 11 [3 Å, 4 Å), class 12 [4 Å, 5 Å) …, until class 18 [9 Å, 10 Å), class 19 [10 Å, 15 Å), and class 20 [15 Å, +∞). The ranges are not equally distributed, but more condensed for smaller values because most of the cases have deviations in the range of 1–5 Å. Figure 6 shows the architecture of an SdA.

The 10 SdAs each have a different number of hidden layers and different number of encoders in each layer. Each of these 10 SdAs was trained using 1/10 of our entire set of training examples. This fraction of training examples was further split into three sections: training (70%), validation (20%), and testing (10%). The training examples were used for both unsupervised learning of the layers of denoising autoencoders (not using the Y or target values) and the fine-tuning after a logistic regression model was added on top of the stack of denoising autoencoders.

For each SdA, the output values in each of the 20 output neurons were treated as probabilities and were at first averaged as:


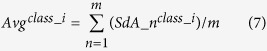


where m equals to 10, which is the number of SdAs; and *class*_*i* is one of the 20 output classes. Based on the averaged probabilities in each class, the class with the largest predicted probabilities was selected as:


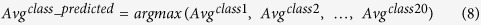


Then a final value is calculated based on the predicted probabilities of two neighboring classes. For example, if class 3 [0.5 Å, 0.75 Å) has the maximum predicted probability 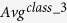, then the final predicted value is calculated as 0.5 + (0.75 – 0.5) * 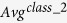/(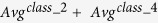). In this way, a real number value is generated based on the two neighboring classes of the class with highest probability.

**Wang_deep_2** uses nine SdAs with different configurations of hidden layers to make predictions first and then combines with an SVM. The nine SdAs take the same input data X, and each generates 20 probabilities. The 20 * 9 = 180 predicted probabilities were then input into an SVM. Our total training data was equally separated into 10 folds, nine of which were used to train the nine SdAs, one for each; and the last fold was used to train the SVM.

**Figure 7:**
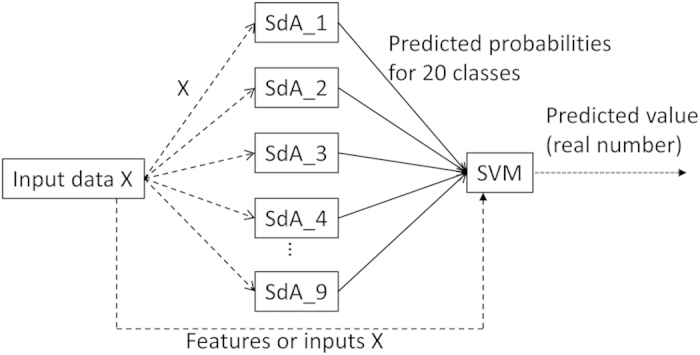
The architecture of Wang_deep_3, in which the original input X was not only input into nine SdAs, but also the Support Vector Machine, together with the 20 predicted probabilities from each SdA.

**Wang_deep_3** is similar to Wang_deep_2, but the same input data X was also input into the SVM, together with the 20 predicted probabilities generated from each of the nine SdAs (Fig. 7).

### Design rationale

Many parameters of SdAs including the number of hidden layers, number of autoencoders in each layer, and training and pre-training epochs can influence the performance of SdAs; and the optimal setup usually can only be found by rounds of trials with different parameters. Because of this nature of SdAs, we designed Wang_deep_1 that makes final predictions based on a simple average on multiple SdAs’ outputs. A support vector machine has been shown to be an efficient machine learning algorithm, therefore, we designed Wang_deep_2 and 3 to combine the popular traditional learning algorithm SVM with novel deep learning architecture SdAs. We think the output values from multiple SdAs that have different configurations can provide useful signatures for the SVM to make predictions. We also designed Wang_deep_3 that takes the original features as input besides the SdAs’ outputs. In this way, the SVM can be informed with both original features and SdAs’ output when making predictions. The design of our four methods includes an SVM only, SdAs only, SdAs plus an SVM with/without double-feeding of original features. In this way, we can have a comprehensive benchmarking between traditional learning algorithm, novel learning architecture, and different ways of combining these two, providing useful insights about how to use deep learning to the protein structure prediction community.

### Global model quality score of prediction

In our methods, the predicted global model quality score (pMQS) for each model was derived from the residue-specific quality scores (i.e., the predicted residue-specific deviations). In particular, for our methods the pMQS for each model was computed as follows:


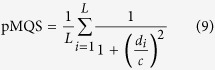


where *L* is the number of residues in a protein of interest, *d*_*i*_ is a residue-specific deviation, and *c* is a constant set to 6.

## Additional Information

**How to cite this article**: Liu, T. *et al.* Benchmarking Deep Networks for Predicting Residue-Specific Quality of Individual Protein Models in CASP11. *Sci. Rep.* **6**, 19301; doi: 10.1038/srep19301 (2016).

## Figures and Tables

**Table 1:** Global evaluation—the overall correlation (R) by PMCC and the weighted mean of Pearson’s correlation (wmPMCC) per target (R.target) for both stage_1 and stage_2 in CASP11.

Method	Stage_1 (selected 20 models)		Stage_2 (best 150 models)	
	R	R.target	R	R.target
Wang_deep_1*	**0.76**	0.67	0.64	0.28
Wang_deep_2*	0.72	**0.69**	0.61	0.28
Wang_deep_3*	0.74	0.68	0.62	0.28
Wang_SVM*	0.74	**0.69**	0.69	**0.35**
ProQ2-refine*	**0.76**	0.70	**0.70**	**0.35**
ProQ2*	**0.76**	0.68	0.70	0.34
MULTICOM-NOVEL#	0.75	0.67	**0.71**	**0.37**
MULTICOM-CLUSTER#	**0.82**	**0.70**	**0.77**	**0.38**
VoroMQA*	0.60	0.58	0.50	**0.38**
FUSION*	−0.04	0.06	0.14	0.08

^*^global score derived from local scores.

^#^global score predicted independently.

**Table 2:** Global evaluation—the 55 target proteins were split into three categories based on their secondary structures: alpha, beta, and alpha-beta.

Method	Stage_1 (selected 20 models)		Stage_2 (best 150 models)	
	R	R.target	R	R.target
Wang_deep_1*	0.56/0.74/0.82	0.55/0.66/0.70	0.74/0.18/0.74	0.19/0.17/0.31
Wang_deep_2*	0.57/0.73/0.78	0.54/0.70/0.71	0.74/0.21/0.70	0.19/0.15/0.31
Wang_deep_3*	0.57/0.73/0.79	0.54/0.70/0.70	0.76/0.14/0.70	0.17/0.16/0.30
Wang_SVM*	0.51/0.68/0.77	0.55/0.68/0.72	0.61/0.22/0.73	0.35/0.23/0.37
ProQ2-refine*	0.71/0.62/0.82	0.54/0.71/0.73	0.65/0.24/0.79	0.35/0.25/0.36
ProQ2*	0.68/0.64/0.81	0.50/0.71/0.71	0.63/0.23/0.78	0.29/0.25/0.36
MULTICOM-NOVEL#	0.40/0.71/0.79	0.51/0.70/0.69	0.42/0.33/0.77	0.46/0.14/0.39
MULTICOM-CLUSTER#	0.61/0.66/0.85	0.46/0.67/0.73	0.53/0.21/0.82	0.31/0.05/0.42
VoroMQA*	0.62/0.57/0.64	0.47/0.67/0.60	0.32/0.48/0.59	0.24/0.28/0.41
FUSION*	−0.18/−0.17/−0.04	0.04/−0.03/0.07	0.10/0.06/0.13	0.08/0.24/0.07

The overall correlation (R) by PMCC and the wmPMCC per target (R.target) were computed in the alpha/beta/alpha-beta categories separately.

^*^global score derived from local scores.

^#^global score predicted independently.

**Table 3:** Global evaluation—the 55 target proteins were classified into two categories: 37 TBM and seven FM proteins.

Method	Stage_1 (selected 20 models)		Stage_2 (best 150 models)	
	R	R.target	R	R.target
Wang_deep_1*	0.77/0.43	0.76/0.56	0.56/0.55	0.27/0.47
Wang_deep_2*	0.76/0.35	0.77/0.58	0.54/0.47	0.27/0.50
Wang_deep_3*	0.76/0.39	0.76/0.59	0.54/0.51	0.27/0.49
Wang_SVM*	0.75/0.39	0.78/0.62	0.56/0.51	0.33/0.54
ProQ2-refine*	0.78/0.45	0.79/0.59	0.64/0.53	0.35/0.46
ProQ2*	0.77/0.47	0.76/0.59	0.63/0.53	0.34/0.46
MULTICOM-NOVEL#	0.76/0.30	0.75/0.57	0.64/0.40	0.37/0.48
MULTICOM-CLUSTER#	0.79/0.50	0.77/0.58	0.67/0.59	0.36/0.49
VoroMQA*	0.62/0.40	0.62/0.57	0.50/0.41	0.39/0.45
FUSION*	0.05/−0.23	0.16/−0.39	0.12/−0.12	0.14/−0.18

The overall correlation (R) by PMCC and the wmPMCC per target (R.target) were computed in the TBM/FM categories separately.

^*^global score derived from local scores.

^#^global score predicted independently.
